# How Children’s Mentalistic Theory Widens their Conception of Pictorial Possibilities

**DOI:** 10.3389/fpsyg.2016.00177

**Published:** 2016-02-26

**Authors:** Gabriella M. Gilli, Simona Ruggi, Monica Gatti, Norman H. Freeman

**Affiliations:** ^1^Catholic University of MilanMilan, Italy; ^2^Faculty of Psychology, e-Campus University of Novedrate (Como)Novedrate, Italy; ^3^University of BristolBristol, UK

**Keywords:** artworks, representation, pictorial theory, theory of mind, intention, exhibition, forgery

## Abstract

An interpretative theory of mind enables young children to grasp that people fulfill varying intentions when making pictures. We tested the hypothesis that in middle childhood a unifunctional conception of artists’ intention to produce a picture widens to include artists’ intention to display their pictures to others. Children aged between 5 and 10 years viewed a brief video of an artist deliberately hiding her picture but her intention was thwarted when her picture was discovered and displayed. By 8 years of age children were almost unanimous that a picture-producer without an intention to show her work to others cannot be considered to be an artist. Further exploratory studies centered on aspects of picture-display involving normal public display as well as the contrary intentions of hiding an original picture and of deceitfully displaying a forgery. Interviews suggested that the concept of exhibition widened to take others’ minds into account viewers’ critical judgments and effects of forgeries on viewers’ minds. The approach of interpolating probes of typical possibilities between atypical intentions generated evidence that in middle childhood the foundations are laid for a conception of communication between artists’ minds and viewers’ minds via pictorial display. The combination of hypothesis-testing and exploratory opening-up of the area generates a new testable hypothesis about how an increasingly mentalistic approach enables children to understand diverse possibilities in the pictorial domain.

## Introduction

An important part of a conception of the pictorial realm is that it encompasses viewers who want to see pictures, and artists who intend their pictures to be seen. Over much of the life-span, people have repeated practice at spontaneous monitoring of pictorial intentions. Eventually, repetition generates representations available to conscious awareness (Karmiloff-Smith, 1992) which then become everyday assumptions which researchers try to identify (Parsons, 1987). The present investigation focuses on how children rely on their theory of mind to make sense of diversity within the pictorial domain, spanning typical and atypical exemplars. The advantage of acquiring a complex theory of mind in furthering an understanding of diversity was one of the central points made by Wellman (1990; see also Myers and Liben, 2012; Hartley and Allen, 2014). Children learn to understand that human beings “use cultural tools for leaving an intentional trace of their communicative and cognitive acts” (Karmiloff-Smith, 1992, p. 139). In the first part of the investigation reported below we tested a reformulation of the proposal that children come increasingly to conceptualize artists and viewers as linked in a common communicative endeavor (Freeman, 2000, 2011). Our reformulation is the strong version of that, namely that during middle childhood, the children go beyond (a) representing artist and viewer as linked separate agents, and (b) integrate the two aspects of agency into a representation of someone who intends both to produce and to display pictures. We test that, then present two exploratory studies which serve an advisory function in how to advance on the test in the next round of research to encompass artists who cheat by displaying a forgery.

Mentalistic reasoning is a way of combining interrelated concepts of belief, desire, emotion, and intention which are integral to an understanding of mind. The child learns to extend mentalistic reasoning to encompass a variety of relations between agents and their world (Bloom, 2002). Agents’ effects on the world are understandable in the light of how agents’ intentions are (a) activated by their desires, and (b) shaped into plans by agents’ beliefs about how to fulfill the intentions (Wellman, 1990). Such reasoning is necessary for understanding artifacts that the agents produce (see Keil, 1989, p. 49 on ‘The need for intention’). Pictures are ubiquitous artifacts. Mentalistic reasoning by viewers is “an appropriate response to the central fact about art: that it is an intentional manifestation of mind” (Wollheim, 1993, p. 134; also Bloom and Markson, 1998; Freeman, 2000, 2008). Artists produce pictures non-arbitrarily, influenced to varying extents by the artists’ conception of viewers’ minds. Artists typically do not just intend to produce pictures but to display them to others. It is only through understanding intentions to display that children can come to understand their own everyday pictorial environment. That is, at some point in understanding the world of pictures, the child progresses beyond (a) a unifunctional concept of the typical artist intending to produce pictures, to (b) a plurifunctional concept of the typical artist intending both to produce pictures and to have them seen. It is at present not known when such insight is acquired and integrated with other insights about the pictorial domain. What is known is that the end-state of development, an untutored adult theory of art, does incorporate mentalistic reasoning centering on intention. Pictures often seem automatically to trigger a search for what the artist intended (Jucker et al., 2014), thereby enhancing the viewers’ liking for the pictures (Jucker and Barrett, 2011). Presumably the increased liking in turn reinforces the search for intention. Indeed, naïve viewers may be too intention-minded. Rosset (2008) formulated a model in which adults habitually monitor others’ actions for evidence of intentions (see also Moore and Pope, 2014) and the monitoring incorporates a positivity bias: when asked to interpret an action, intentions are ascribed more than they should be. It is true that it may be a mistake for viewers to rely too heavily on what artists seem to intend, because some artist intentions may be limited as guides to pictorial interpretation (Beardsley, 1981, 1982; Davies, 2013), and artists may err in their suppositions about how viewers react to the art-display (see Salfner and Voigtmann, 1999). However, identification of artist intentions is useful to viewers even if only to serve to generate useful heuristics for interpretation. A prime task for research is to identify how an understanding of artists’ intentions develops.

As is usual in research on mentalistic reasoning, it has been found that important advances in monitoring pictorial intention occur in the preschool years. Preschoolers, when they find a picture unclear, tend to infer what the artist may have intended (Browne and Woolley, 2001). Children as young as 30 months of age spontaneously monitor an artist’s gaze while she draws an object; and when taught a name for the drawing (“This is a spoodle!”) children map the novel word to the real object the artist had apparently intended to draw (Preissler and Bloom, 2008), see also Preissler and Bloom (2008) on 2-years-old, and Salsa and de Mendoza (2007) and German and Johnson (2009) on 3-years-old. Thereafter, a mentalistic stance on depiction widens to incorporate further insights into agency (Gelman and Ebeling, 1998; Callaghan and Rochat, 2003, 2008; Freeman, 2008, 2011; Hartley and Allen, 2014). Perhaps it is not until at least 7 years of age that children undertaking pictorial interpretation may normally consider the mind of the viewer in relation to the mind of an artist (see Golomb, 1992). Accordingly, we should look to middle childhood to identify the foundation of plurifunctional thinking about intention. We followed the suggestion by Freeman and Allen (2013) of setting puzzles which involve intentions that are contrary to the typical possibilities in the pictorial domain.

Study 1 tested the hypothesis that middle childhood encompasses when children replace (a) a unifunctional conception of an artist as someone who produces a picture, with (b) a plurifunctional conception of an artist as someone who both intends to produce and to display a picture to others. A new method was needed. We adapted the foundational false-belief experiment in theory of mind in which an agent hides something which is subsequently found and removed by someone else. Children watched a brief video in which an artist atypically hides one of her pictures from view, then a friend finds the picture and, unbeknownst to the artist, thwarts the artist’s intention by putting the picture on display in a museum (in Italy, *museo* covers both museum and gallery as in The Tate Gallery and the Museum of Modern Art). The child was asked questions, amongst which were three target questions of (a) what people would think of someone who hid her own beautiful picture, (b) whether it is *important* for an artist to display her picture, and (c) whether it is *necessary* for an artist to display her picture. We predict that 5-years-old would unifunctionally judge that the artist had fulfilled her role simply by producing the picture. Ten-years-old would plurifunctionally judge that artists are expected to bear in mind the needs of viewers. An open question is whether the 8-years-old would resemble the younger or older groups.

Study 2 investigated children’s conception of what makes a picture worth displaying, asking whether (a) beauty, (b) authorship, or (c) critical opinion were most important in judging a picture. The firm prediction from Parsons (1987) is that 5-years-old’ thinking is dominated by the question of how beautiful a picture is. Older children can safely be predicted to move toward a plurifunctional judgment, encompassing both which artist produces the picture, and experienced viewers’ opinion that the picture is worth displaying. This aspect of the study is exploratory, designed to open the area where, interestingly, the typical rate of development has not yet been identified.

Study 3 involved a converse of hiding something, namely atypical display of something misleading. The question is whether deliberate display of a forgery disqualifies an artist from a claim to the role, in a similar way to refusal to exhibit an original. Forgery poses one of the most complex topics for naïve pictorial reasoning that accounts of the domain currently encompass (see Bullot and Reber, 2013). We asked children what is wrong with a forgery, and whether they themselves would put a forgery on display. Children’s reasons for their judgments should suggest whether an explicitly mentalistic conception of an authentic display piece arises before the end of middle childhood and facilitate the formulation of a testable hypothesis.

To conclude, the investigation below involved three distinct types of study in a repeated-measures design. Questions were formulated within semi-structured interviews, many of them required yes/no answers, thus facilitating quantitative analysis where the alpha level can be set very high. Another essential part of the approach is to identify mentalistic reasoning irrespective of whether the child answered a question affirmatively or negatively. The overall approach thus employs frequency data and qualitative data in complementary fashion (see Freeman and Parsons, 2001) to identify whether, in the 5-years span of middle childhood, a shift toward mentalistic reasoning characterizes children’s approach to the pictorial domain.

## Study 1: Does a Picture-Producer’s Role Involve Displaying Pictures?

### Participants

We interviewed 30 Italian urban children: 10 5-years-old (six girls, four boys), 10 8-years-old (four girls, six boys), and 10 10-years-old (six girls, four boys). The studies were performed under the ethical guidelines of the American Psychological Association and the Italian Association of Psychologists. In accordance with the APA (2010), the participants’ parents gave their written informed consent to the experimental procedure. Anonymity of data was also guaranteed. There were no conflicts of interest that could have prejudiced the conduct or presentation of this investigation at any stage.

### Procedure

Children began with a session designed to establish rapport and to familiarize the children with the topic of pictorial judgment. First, the experimenter talked informally with the children, asking about their tastes in to drawings, paintings, and color-preferences. The interviewer specified an interested in what the children think about some artistic issues, and that there are no right or wrong answers to the questions that will be asked. Children were then interviewed individually in a quiet room for about 15 min.

The interview began with viewing a purpose-made silent video (duration: 2.10 min) in their classroom. There were two people in the video, neither of whom was a professional actress. In scene 1, a woman sits at a table, painting. She then puts down her brush, looks at her picture with a satisfied expression, then hides it in the drawer of the table, and leaves the room. In scene 2, another woman enters the room and looks around. She opens the drawer, and finds the picture. She looks at it, smiles, and exits taking the picture with her. In scene 3, a building with a sign “Museum” on it is shown. The woman enters and hangs the picture she took from the drawer on a wall in the museum.

The following six questions in colloquial Italian were asked in randomized order:

To be identified as an artist, is it necessary to exhibit your works in a museum?

Must the artist show her works to other people?

Is it important for the artist to exhibit her pictures?

In order to become famous, does an artist have to exhibit her works in a museum?

If I paint a very beautiful painting and I keep it hidden at home, will people think I am an artist or not?

If you create a work of art and you do not show it in a museum, are you an artist or not?

### Results

The data of interest in this study were only whether children answered yes or no, not the details of the reasons given by the children. Note that each question wording required a yes or no answer, but the children were free to add spontaneous comments about their reasoning. If the added comments obscured the child’s position on a question, the interviewer said that he understood and again asked the question in the manner that someone would who was checking that they indeed understood. For example, after the question “In order to become famous, does an artist have to exhibit her works in a museum?” one 10-years-old child replied: “I do not know, the museum is important, and an artist must exhibit his work there, but can also put it on the internet, or in the street.” The researcher then asked: “So do you think the artist must set out her works in a museum?” and the child replied “No, not only in the museum but also in other places.” Accordingly, that child’s answer was scored as No. Assurance was needed that the answers were consistent with the question; therefore the children’s verbalizations were analyzed by three judges. They conducted a preliminary individual analysis on each child’s answers, and then, where necessary, discussed the matter until they reached a unanimous decision (Losito, 1993). Note that the interviewer and judges worked blind to the data-plan used for Section “Results” of the present paper; and the person who drafted that plan and the account worked blind to any details of the data-emergence.

The data-analysis below is quantitative, with qualitative issues confined to illustrative status in examples of answers given below for the purpose of information to show how children were expressing judgments.

With Bonferroni correction, an alpha level of 0.008 was set. The two older groups maintained that artists *must* show their pictures to others (19/20 children, binomial *p* < 0.001, whereas the youngest group denied it by a small majority (6/10 children, binomial chance). The results were identical for a randomly inserted weaker variant question in which it was not asked whether display was necessary but rather whether display was *important*. The data are set out in **Table 1**. The results were identical for the video-related question of whether other people will think that an artist who keeps a beautiful painting hidden is an artist. To give an indication of the reasoning of the most articulate of the children, the 10-years-old, three examples are: “If you paint a beautiful picture and you keep it hidden at home, you are not an artist for others, because artists show their pictures.” Again: “To become an artist a person must show his works of art to the others, if he keep them hidden at home, he will never become an artist because […] the others are the ones who can tell whether he is an artist or not.” And again: “For example, we went to the seaside and we saw on the street one guy who was painting some poster with spray paint and then he was selling them. He was an artist.”

**Table 1:** Frequency of answers in Study 1.

	Age group (years)		
	*5*	8	10
**In order to be identified as an artist, is it necessary to exhibit your works in a museum?**			
Yes, it is necessary for an artist to exhibit in a museum if they are to be considered an artist	4	5	7
An artist has to exhibit but not only in a museum, even in other places	6	5	3
**Must an artist show her works to others?**			
Yes, it is necessary for an artist to show his works to others	4	9	10
No, the artist is an artist even if he doesn’t show his works	6	1	0
**Is it important for an artist to exhibit?**			
Yes, it is important for an artist to exhibit his works to others	4	9	10
No, it is not important for him to exhibit his works to others, it’s	6	1	0
**To became famous, does an artist have to exhibit her works in a museum?**			
Yes, a famous artist exhibits in a museum	7	7	10
No, a famous artist exhibits not always in a museum but also at home or on the roadside	3	3	0
**If I do a beautiful painting and keep it hidden at home, do people think I‘m an artist or not?**			
If I paint, I am an artist even if I don’t exhibit	6	1	0
If I am a true artist I have to show and exhibit my works	4	9	10
**If you create a work of art and don’t show it in a museum, are you an artist or not?**			
If I create a work of art I must exhibit it in a museum to be considered an artist	4	7	8
If I create a work of art I don’t have to exhibit it in a museum, to be considered an artist I must be skilled	6	3	2

In sum, whichever of the three target questions was asked, the two older groups had integrated the intention to display with that of production. It will be economical to defer to the discussion the question of whether the use of systematically varied repetition of questions bears on how strong those data might be.

Scanning down the columns of **Table 1**, it is evident that the 10-years-old were unanimous for 4/6 questions asked, and showed a majority of 7/10 and 8/10 for the remaining two questions. A score of only five deviations from unanimity over 60 answers exceeds expectations of finding firm effects. Clearly, for the children, whilst it is desirable to put a picture in a museum, all agreeing that it is an indicator of fame, a museum is seen as a vehicle for display not as end in itself. Here, are three representative extracts from the older children to illustrate the type of data sustaining the above summary results on their unanimity: “It is not necessary to show them in a museum, the artist can also show them at home, he can invite some people at home…the important thing is to show them.” Again; “If you want to become famous, you must show them … but it depends: if you want to show them, all right, if you want to show only part of them, all right…it’s you who decides.” And again: “It is not always necessarily a museum, it is possible to exhibit also in an art-gallery or in a private exposition…even in the street to show art, to show how beautiful art is, so that more people maybe learn about art. It is to promote art.” It appears that here a basic criterion for being an artist concerns the gaze of others, independently of where the communication from artist to viewer actually occurs. Turning now to the 5-years-old, it is evident that their answers are far from unanimity, always being a 6-4 or 7-3 split. The 8-years-old reassuringly present an intermediate state of affairs between the 5- and 10-years-old results.

To sum up, the results are as predicted from the strong version of Freeman’s position. That is, the evidence from the very first trial show that children during middle childhood the children integrate artist and viewer as into a representation of someone who intends both to produce and to display pictures. What we could not have predicted was the age at which that integration was evident.

## Study 2: Making an Informed Judgment About Typical Possibilities

The next study focused on typical possibilities in the pictorial domain. One topic centered on what is decisive in judging a picture worthy of display, over the span from its origin of production, its quality as a product, and the subsequent mentality of art-critics. Critics are supposed to make up their minds on what is deemed worth exhibiting. The second topic highlighted the question of beauty that is a criterion in everyday naïve esthetics (Freeman, 2004; McManus, 2011). Asking questions about pictorial beauty been used to identify a developmental trend (Parsons, 1987), from the age of 5 years onward (Freeman and Parsons, 2001). The trend is particularly useful for normative purposes to check whether a sample seems to be typical or precocious.

### Procedure

After a short break, the child was again interviewed back in the same quiet room for about 15 min. Children were asked five randomized questions (note that the Italian superlative was used in the first three questions listed below, but acts less radically than in English, i.e., is an intensifier of X with the sense of ‘extremely X’).

What is extremely important when judging a work of art? Is it how beautiful it is?

What is extremely important when judging a work of art? Is it who made it?

What is extremely important when judging a work of art? Is it the judgments of art-critics?

Does a work of art made by a famous artist have more value than one made by an unknown person?

Is beauty the only thing that matters in judging a work of art?

The method of conducting the interview and coding of responses is the same as in the previous study.

### Results

**Table 2** contains two cells where one of the questions was omitted, because the 5-years-old did not know what the term ‘art-critic’ meant. Setting those cells aside, scanning the top rows of **Table 2** reveals that all the 5-years-old favored beauty as a criterion for judgment (binomial *p* = 0.001, beyond the Bonferroni-corrected alpha of 0.01), reassuringly in line with Parsons (1987) insistence on young children’s prioritization of beauty. As one child commented “It is art because it is beautiful.” In contrast, 9/10 of the 10-years-old identified authorship as a criterion for judgment (binomial *p* = 0.001), and divided 7-3 on beauty (binomial *p* = 0.50). Thus a reversal occurred between the youngest and oldest groups. Again, the 8-years-old were intermediate and sometimes made complex judgments, e.g., “When you judge a painting the beauty is important. First of all you see if it is beautiful or ugly. Then it is also important to know who the author is.” Using beauty as a criterion, the sample appears developmentally congruent with that of Freeman and Sanger (1995) for this most common of esthetic considerations.

**Table 2:** Frequency of answers to What is most important when judging a work of art?

		Age group (years)		
		5	8	10
Beauty	YES	10	6	7
	NO	–	4	3
Authorship	YES NO	–	6	9
	NO	–	4	1
Judgments of art-critics	YES NO	–	8	8
	NO	–	2	2

The 10-years-old were almost unanimous (9/10, binomial *p* = 0.001) that it is extremely important to know who was the painter when judging a picture: “If you don’t know the story of that person, if he was sick…, you cannot understand the story of the painting.” Turning now to the role of art-critics, in the older two groups 16/20 children (binomial *p* = 0.006) endorsed critics’ importance even given the considerations of beauty and of authorship: “When you have to evaluate an artwork, it is important to ask advice from art critics …if you do not know it, the picture loses a part of its value.” Art critics were described as experts, authorized personnel who can transmit judgments: “Knowing what the art experts think is important because they understand better than you…they can help you. I would surely listen to them”; and “…they are experts about these things and they know a lot more things than me.” The results are not quite as strong as the unanimity that was evident in Study 1 on the basic concept of a viewer. Presumably, extension to informed critical viewer judgment is still in process of formation.

Finally, authorship was again asked about as shown in **Table 3**. The 5-years-old almost all (9/10, binomial *p* = 0.001) denied that it mattered to the value who produced an artwork, but again the older groups lacked unanimity. And when a forced question-form was used, asking whether beauty is all that matters in judging a picture, again the youngest were almost unanimous in judging that it was all that matters (9/10, binomial *p* = 0.001), and the older groups were divided. Examples of older children’s reasoning is as follows: “Knowing what the art critics think is important, because with a positive judgment a picture increases its value, whereas with a negative judgment its value decreases.” Again: “Yes, knowing who the author is… yes, because it lets you know something more about the picture…it gets across to you also the story of the picture, since you could also ask for information from the author. Each painting is unique also because the artist transmits his narrative through the painting.”

**Table 3:** Frequency of answers to Is a work of art by a famous artist more valuable than one made by an unknown artist? and Is beauty the only thing that matters in judging a work of art?

		Age group (years)		
		5	8	10
A work of art made by a famous artist has more value than one made by an unknown author?	YES	1	8	7
	NO	9	2	3
Is beauty the only element that matters in judging a work of art?	YES	9	8	7
	NO	1	2	3

To sum up, the results are as predicted from Parsons’ work on children’s understanding of art in that the youngest children’s thinking was predictably dominated by the question of beauty regardless of authorship; and three-quarters of the older children’s reasoning had developed beyond that. What we could not have predicted was the degree of diversity of thinking in the older children.

The next study forms a converse of the first study, whereby instead of hiding an original picture an artist puts a forgery on display. The technique is to tell the children of a skilled forger, Van Meegeren, then to interview the children about what is wrong with forgery. The aspect of interest is whether the children give unqualified moral reasons (forgery is wrong in some respect) or whether they give mentalistic answers in terms of the effects of forgery on viewers’ minds. A conception that it is authentic products that can be displayed in a museum becomes incorporated into a naïve theory of art (Newman and Bloom, 2012; see also Cavanagh et al., 2013; Seeley, 2013). Young children condemn passing off someone else’s originality as one’s own even if the children themselves like the copy (Frazier and Gelman, 2009). The assumption yet again is that there will be a developmental shift evident after the age of 5 years in the direction of mentalistic reasons being given by the children for their judgments.

## Study 3: The Production of Falsity in Picture-Production and Display

### Procedure

The children were shown images of two paintings (printed in color in A4 format), one original, “The Milkmaid” by Vermeer, and the other one forged (*falso*), “Supper at Emmaus” by Van Meegeren. While showing Vermeer’s painting, children were told about the peculiarities of the artist’s style. In showing the forgery, children were told in non-evaluative language about Van Meegeren’s history, highlighting his intention to copy perfectly the style of another artist, to pretend to be him, and the fact that once the truth came out the picture then lost monetary value. Ten-minute interviews were then individually given, in a quiet schoolroom, with four randomized questions:

Is it important that a museum exhibit the original works of an artist?

Is a fake equally as valuable as an original picture?

Would you show a fake in a museum or an art gallery? Why?

Why is a fake not OK? What is the problem with a fake?

The method of conducting the interview and coding of responses for the first questions was the same as in the previous studies. In addition, for the last two questions, the judges were asked to undertake a content analysis and to create two modal categories, afterward to assign each answer to one or the other category.

### Results

With Bonferroni correction, an alpha level of 0.01 was set. We begin with the two questions that served as a check for whether the children had understood the scenario. The children had been told that the picture had lost its monetary value when found to be a forgery, and when the question ‘Is a forgery equally as valuable as an original picture?’ was asked as a control question, the judges were unanimous that all 30 children passed (binomial *p* = 0.001) as shown in **Table 4**. Secondly, every child affirmed that a museum must show only originals (binomial *p* = 0.001), and the switch from affirmative to negative here rules out simple response-bias as a potential confounding.

**Table 4:** Frequency of answers to questions in Study 3.

	Age group (years)		
	5	8	10
**Is it important that a museum exhibit artists’ original works?**			
Yes, the museum must exhibit only original works	10	10	10
No	0	0	0
**Is a fake as valuable as an original picture?**			
Yes	0	0	0
No, the original is more important	10	10	10
**Would you show a fake in a museum or an art gallery? Why?**			
I would exhibit original paintings because the public might lose trust in the museum	1	8	6
I would exhibit original paintings because it’s wrong to cheat visitors	9	2	4
**Why a fake is not ok? What is the problem with a fake?**			
Faking it is wrong because it’s cheating to copying what the others made	10	7	6
The forger doesn’t prove his ability and creativity	0	3	4

Accordingly, given the response agreements, it is now feasible to ask whether the children all give the same reason for why museums avoid forgeries. Is it merely because originals are worth more? Children’s answers were clear and the judges easily picked out the same two categories. One category was confidence in the museum that chooses what to display, e.g., an 8 years-old said, “The museum is an important place and the paintings that are there are important and chosen to show them to visitors and those who go there trusting in the museum.” The second category concerned the rightful display of only original paintings, representative of the artists’ creativity. **Table 4** reveals a cross-over in the reasons offered. Of the 5-years-old, 9/10 took up a moral position, asserting that it was wrong to cheat viewers, whilst the remaining child took up the mentalistic position that it might cause viewers to lose trust. In contrast, the 8-years-old split in the reverse direction by 2-8 (the cross-over is significant at *p* < 0.01, Fisher test). Clearly some important development was occurring. However, in that light we do not know why the 10-years-old split nearly 50-50, at 6-4. One looks to the final remaining question for possible illumination. The question ‘What is wrong with a forgery?’ proved interesting in three respects. First, not a single child answered in impersonal terms of the loss of monetary value compared to an original, and the majority, 23/30, confirmed that what is wrong with a fake is that it involves cheating (binomial *p* = 0.003), with all 10 of the 5-years-old asserting that anti-cheating moral position (binomial *p* = 0.001). Secondly, the 8-years-old again were less than unanimous but this time with the majority going in the 5-years-old direction with 7/10 asserting a moral position, so no great development is manifest. The 10-years-old were again split, with 6/10 asserting the moral position, so the 10-years-old split on the previously tabulated question looks less aberrant. Thirdly, the seven non-moral answers from both the older groups, a third of the older groups, are interesting. All seven children asserted that forgery was wrong because forgers disadvantage themselves by not being able to display their skills as an original artist. That reason is of particular interest because the story had made explicit how the forgery had indeed enabled the forger to show skill with the paintbrush. To the best of our knowledge, that aspect of an early view of the importance of originality in artwork has not been documented in the psychological or art-education literature (though see Wolz, 2014, for a pioneering study involving perception of talent in original artists and forgers).

To sum up, the results are exploratory and contain a diversity of children’s reasons for their judgments. The use of a transgressive act of picture-display, a forgery, provoked the diversity in line with the suggestion of Freeman and Allen (2013). It will be interesting to use the results to compile a set of forced-choice options to present to children.

## Discussion

In Study 1, three variants of a target question probed whether artists intended to show their work to others. The finding was that 8-years-old had replaced (a) a unifunctional categorisation of an artist simply as a picture-producer, in favor of (b) a categorisation of an artist as an agent who is linked to viewers. Previously, Maridaki-Kassotaki and Freeman (2000) had identified the emergence of a critical mentalistic theory of art unifying picture-production and picture-display at preadolescence. The new finding suggests that one should look to even younger children for a conceptual transition. Where Freeman (2004, 2011) had identified the conceptual challenge facing children, we can now add that 8-years-old rise to the challenge by firmly integrating the role of viewer with the role of artist. Study 2 investigated whether (a) beauty, (b) authorship, or (c) critical opinion were most important in judging a picture. The results confirmed the focus on pictorial beauty by 5-years-old predicted from the literature inaugurated by Parsons (1987). Beyond that age, the study served to open the field of possibilities of developmental change, and here, respect for the minds of art-critics emerged in 16/20 older children (*p* < 0.001, binomial), pertaining to one of the highest levels of mentalistic engagement with pictures identified by Bullot and Reber (2013). Again, the majority of 8-years-old were revealed to be mentalistic reasoners. Study 3 found a cross-over in reasons given for why forgery is unacceptable. Five-years-old were unanimous that it is wrong to cheat, whilst the 8-years-old were incorporating a mentalistic concept of viewers losing trust in the authority of the museum. To the best of our knowledge, documentation of that acquisition is not to be found in the experimental literature. Nor is the sign, in a third of the older groups, of awareness of forgery as hampering the artist in demonstrating artistry, despite the preceding briefing on van Megeeren having mentioned his assiduity and skill.

Before discussing implications of the evidence, it is essential to evaluate the quality of the evidence. It will be economical to focus the questions whenever feasible to the particular study they bear on most cogently, rather than repeat the discussion for every detail in the repeated-measures design.

### Sample Characteristics

The most general question of all is whether the sample of children had any developmentally atypical characteristics? The concern is whether the complex cultural context within which the sample grew up might make the data normatively of limited value. Might the children have been culturally led into an atypical developmental pathway? The sample was made up of urban Italian children in a country with the richest pictorial art collection in Europe. The context of the investigation is thus one of familiarity with art display. We suggest that the consideration is plausible, but two pieces of evidence ameliorate the concern. First, there is no evidence that Italian children become pushed to any conceptual extreme by their exposure to art: thus, Ruggi and Gilli (1998) found that Italian children still took their criteria for what is beautiful and good from nature instead of from pictures. Secondly, there was the focus on beauty predicted from previous work in other cultures, with both a pictorially representative sample (Parsons, 1987) and a pictorially disadvantaged sample (Freeman and Sanger, 1995) That focus on beauty was shown in the 5-years-old and held up under forced-choice questioning in Study 2: beauty was all-important and authorship was irrelevant. The conclusion is that there seems to be no normative disadvantage in using the sample. On the contrary, there is the advantage that Italian children find it conversationally natural to talk about art and the display of art without floundering. That has three consequences. First, it is easy to establish and maintain rapport over the topic. Secondly, children readily acquire a pictorial vocabulary with which to formulate their judgments, e.g., a distinction between pictures in general (*disegni, illustrazione*) and display-pieces (*quadri*). The availability of the distinction makes it easy to focus on display, which was the target of Study 1. The distinction appears also in Spanish, which would perhaps be first choice for systematic replication. Finally, as was noted previously, it was never necessary to rephrase any of the questions.

### Sample Size

The question is whether the trade-off between sample size and lengthy testing may have been too one-sided: sample sizes of 10 per age-group were small. Yet the data in the primary study, Study 1, were compelling in terms of level of probability with Bonferroni-protected *p*. In particular, given that only 1/20 children in the older groups was an exception to unanimity that an artist must display pictures, we suggest that the data were strong enough for their purpose. Conventionally, *p* < 0.001 is generally acceptable. It would be encouraging if future research found that small samples were adequate to expose robust effects at the level of individuals.

### Individual Consistency under Repeated Questioning

In any investigation which relies on extended work with children, the hope is that each child remains in a fairly stable state as a data-generator (e.g., not becoming tired or self-conscious). In that light, there are two procedural points about any investigation, two advantages to repetition and two dangers to be tested for. We shall take these six considerations in order.

One procedural points is to avoid blurring the distinction between new and old questions, so that it should always be discursively clear when one is opening a new topic without any implicit pressure to recall what has already been said in the interview. The other procedural point is to introduce changes in topic if feasible to maintain interest and again to help minimize carry-over effects. Both of these were incorporated into the present investigation, by (a) never having literal repetition of any question, and (b) interpolating Study 2 on non-mental states between the two target-issue studies. We propose that the present investigation conforms to those two requirements.

The first advantage to a long procedure lies in not having repeatedly to establish rapport. It is easy to monitor rapport, once it is established, and with increasing acquaintance with the child it is easy to maintain rapport. We noticed no failure in the process, in particular no drop-out threatened to occur. The second advantage lies in the stability of the sample: is that there is no need to check whether the introduction of each new individual into the investigation is adding noise to the corpus of evidence by virtue of their developmental status or their context (e.g., noisier classroom or anxiety-ridden school). We conclude that the present investigation has those advantages.

We finally consider the two dangers: (a) that an individual’s answers may be unstable under repeated questioning, and (b) the converse danger that an uninformative uniformity is inculcated. Let us take these in order. Rose and Blank (1974) proposed that children, if they think that they are being asked a similar question twice because they had given the wrong answer before, will be liable mechanically to change their answer (see Siegal, 1997, for a discussion of five alternative explanations of the effect). Yet in Study 1, the target data went in the opposite direction to the Rose-Blank effect: all the children gave their particular answer each time over the three related questions. Note that the consistency necessarily means that the children gave their particular answer on the very first time of asking, so the judgment cannot be attributed to any unwanted order-effect. However, there is another opportunity to raise the repetition question, in Study 2. The older children’s judgments were marked by unpredicted diversity. Might that diversity be an aspect of the Rose-Blank instability of answering, only this time at the sample level rather than within an individual’s sequence of answers? Yet there was less reiteration than in Study 1; and if reiteration was not a problem in Study 1, there is no apparent reason why it should obtrude with two of the groups in Study 2. That leaves two possibilities. On the one hand, maybe the diversity is a sign that children were in the process of taking into account disparate considerations, much as Freeman (2000, 2004, 2011) has argued. On the other hand, maybe there is potential unanimity which is obscured by the procedure of enquiring into typical possibilities. Certainly the analytic power of investigating atypical possibilities was the rationale in theory of mind research, leading to the focus on false beliefs. Either way, the primary purpose of Study 2 was only to delimit an area of testable possibilities in children’s conception of display.

Lastly, in Study 2, the children might recall Study 1 and come to infer a theme and a core interest of the adult. Might that lead to children giving mechanically compliant answers? Study 1 had been centered on picture-display, so in Study 2 we deliberately avoided posing any question about the importance of display. There was no basis for a desired answer to be inferred about whether it mattered if a picture was beautiful or not. That enabled us to document the predicted beauty-centered criterion in the younger children. In Study 3 after denying the value of a forgery, all the children affirmed that a museum must show only originals. That could be compliance. However, that affirmation was merely a check on whether the children understood the problem. The actual data, the reasons they gave for their judgment, had no basis in priming form Study 1. So those data are suitable to serve an advisory role for the next step in research. We conclude that we can rule out simple compliance as a complicating factor.

In sum, while no method can be perfect, we conclude that the present approach is safe from the major problems of repeated interview, and that it benefits from the commonest precautions. In particular, the crucial target data from Study 1 were consistent right from the very first question, so could not have become weakened by uninformative instability or compliance. It is straightforward to try replication on that. For the rest of the study, we do not suggest attempted literal replication: it would be far more informative to do systematic replication.

### Setting the Novelty of the Approach in Context

The most general question is whether it is worth continuing the present approach in future. To answer that it is necessary to consider (a) what continuity there is with previous approaches, set against (b) what innovation is in the present approach. It seemed reasonable to use adopt a common procedure which slowly increased children’s involvement. The procedure began with a simple scenario which breaks the norm of an artist’s role in displaying pictures by having an artist hide a picture. That formed a single target-question test, rather in the economical way that a false-belief test does, involving some mismatch with an expectation. That target test was preceded only by the rapport phase. The ease of establishing rapport over the topic means that there is no serious doubt over the complementary worry that children might not have warmed up. The final target study continued the theme of norm-breaking by focusing on displaying a forgery. The data-processing was refocused on whether or not children mentioned any uncued mentalistic considerations. The essential innovation was to center each study on the concept of pictorial display. Whilst the role of an artist is naturally concerned with picture-production, the role of a viewer can only be carried out once a picture is put on display. So, if one is to study the child’s conception of relations between picture, artist, viewer, and their world (Freeman, 2008), it is important to investigate the child’s conception of a relation between artist and viewer whereby an artist hands over a picture for display which then makes it possible for someone else be a viewer. We conclude that the method seems suitable for extension to child’s conception of what intentions a viewer should have if they wish to understand the minds of the artists, and their pictures.

### Development of the Method

In that light, it is worth raising the question whether the method should continue to avoid giving mentalistic cues. The protocols were made to contain as few explicit verbal mentalistic cues as feasible, as did Maridaki-Kassotaki and Freeman, 2000 when investigating spontaneous mentalistic conceptions of a pictorial display-piece. It is empirically possible that the children have deeper mentalistic formulations which might be activated by cues. It is, however, important to study spontaneous uncued conceptions because those provide an immediate point of entry for art-educational intervention. There are psychological lessons to be learned from art education attempts to promote and influence children’s theory of art (Freeman, 2008; Freeman and Allen, 2013). We suggest that a useful research strategy is to employ the following converse methods. With older children’s spontaneous reasoning, it is worth documenting spontaneous reasoning as widely as possible with cues. With preschoolers, where the salience of pictorial intention is documented (Bloom and Markson, 1998), it is worth concentrating on methods designed to identify implicit conceptions below the level of awareness where the preschoolers cannot access them for themselves (much as has been done for false-belief tests: Freeman et al., 1991; Clements and Perner, 1994; Freeman and Lacohée, 1995; Clements et al., 2000; Schneider et al., 2015). Presumably one way forward is to continue the adaptations of a false-belief design as in Study 1 which delivered the crucial yes/no data on children’s conceptual integration of artist and viewer.

### Theoretical Implications: Mentalistic Reasoning

A viewer may be a passive beholder of a picture or an active agent attempting to become involved in the artist’s experience. The issue of artists’ and viewers’ intentions is hugely important as an aspect of agency. Note that mentalistic concepts are interdependent: one should consider an intention to do something along with (a) a desire to do something, and (b) a belief that something is worth the attempt. The present approach is necessarily not solely about intention to the exclusion of other components of mentalistic reasoning. However, the concept of intention is fundamental because intention is at the origin of action-plans. The agent who implements an action plan and creates an artifact is a key component of everyday reasoning about who has priority in ownership of the artifact (Levene et al., 2015). There is a suggestion from Study 3 about how 8-years-old may distinguish between authentic pieces and forgeries. The issue of forgery becomes interesting here because creation is divided between two agents. One agent creates the original and another agent creates the forgery. The two agents have distinct intentions toward a viewer. We predict that 8-years-old will segregate their intentional concepts of artist and forger, judging that an artist who does not display her picture cannot be regarded as an artist but that a non-displaying forger is still a forger.

A second developmental question is whether young children put too much weight on intention as though it dictated how a picture must be interpreted. Bloom and Markson (1998) argued that for preschoolers, if a picture-producer has the intention of representing a balloon by a circle and line, that shape cannot then represent a lollipop. For preschoolers, intention thus restricts pictorial possibilities. We suggest that it is timely to investigate when the restriction is overcome, and an understanding of intention then becomes a resource for children. It has long been recognized that even very young children have the experience not only of forming clear pictorial intentions, but of changing intentions during the course of drawing (Freeman and Adi-Japha, 2008).

Such issues concern the wider theory in which the present work is embedded. Theory of mind research has energized research into topics in adjacent domains (e.g., Fink et al., 2015, on friendship; Freeman et al., 2000, on numeracy). Pictorial reasoning emerges as a distinct domain which becomes developmentally enriched by mentalistic reasoning. There are developmental differences between the theory of mind domain and pictorial reasoning. Even in preschool, there are empirical differences between passing procedurally matched false belief tests and outdated picture tests (e.g., Peterson and Siegel, 1998). It appears that after the age of 5 years, children begin the conceptual work of incorporating a representation of the viewer into that of an artist. Artists are supposed to show their work to others, and the work is supposed to be genuine lest viewers lose trust. The process of such acquisition may be characterized as one of moving from a fixation on pictorial beauty and moral integrity to a mentalistic conception of the art domain. Possibly, ‘Children might call a picture that looks like a bird “a bird” not merely because it looks like a bird, but because its appearance makes it likely that it was created with the intent to represent a bird. In general, appearance – and shape in particular – is seen as an excellent cue to intention’ (Bloom and Markson, 1998, p. 203). In that respect, there opens a two-way relation between theory of mind and theory of art: visual communication (Freedman and Stuhr, 2004). Eight-years-old remain an intriguing group in the throes of acquisition, where alongside making predictions about them it is important to discover hitherto-hidden facets of their reasoning. Whereas, Study 1 and half of Study 2 tested hypotheses, the other half of Studies 2 and 3 were steps in exploring the range of possibilities that the domain affords.

## Author Contributions

All authors listed, have made substantial, direct and intellectual contribution to the work, and approved it for publication.

## Conflict of Interest Statement

The authors declare that the research was conducted in the absence of any commercial or financial relationships that could be construed as a potential conflict of interest.
